# Development and multi-cohort validation of a machine learning-based simplified frailty assessment tool for clinical risk prediction

**DOI:** 10.1186/s12967-025-06728-4

**Published:** 2025-08-15

**Authors:** Jiahui Lai, Cailian Cheng, Tiantian Liang, Leile Tang, Xinhua Guo, Xun Liu

**Affiliations:** 1 Department of Nephrology, Third Affiliated Hospital of Sun Yat-Sen University, 600 Tianhe Road, Guangzhou, 510635 Guangdong China; 2 Department of Cardiology, Third Affiliated Hospital of Sun Yat-Sen University, 600 Tianhe Road, Guangdong 510635 Guangzhou, China; 3 Department of Rheumatology, Third Affiliated Hospital of Sun Yat-Sen University, 600 Tianhe Road, Guangdong 510635 Guangzhou, China

**Keywords:** Frailty assessment, Machine learning, Chronic kidney disease, Cardiovascular risk, Mortality prediction, Risk stratification, XGBoost

## Abstract

**Background:**

Frailty significantly impacts health outcomes in aging populations, yet its routine assessment remains challenging due to the complexity and time-consuming nature of existing tools. This study aimed to develop and validate a clinically feasible, machine learning-based frailty assessment tool that balances predictive accuracy with implementation simplicity in real-world clinical settings.

**Methods:**

We conducted a multi-cohort study leveraging data from the National Health and Nutrition Examination Survey (NHANES, *n* = 3,480), China Health and Retirement Longitudinal Study (CHARLS, *n* = 16,792), China Health and Nutrition Survey (CHNS, *n* = 6,035), and Sun Yat-sen University Third Affiliated Hospital CKD cohort (SYSU3 CKD, *n* = 2,264). Through systematic application of five complementary feature selection algorithms to 75 potential variables, followed by comparative evaluation of 12 machine learning approaches, we developed a parsimonious assessment tool for predicting frailty diagnosis, chronic kidney disease progression, cardiovascular events, and all-cause mortality.

**Results:**

Our analysis identified a minimal set of just eight readily available clinical parameters— age, sex, body mass index (BMI), pulse pressure, creatinine, hemoglobin, and preparing meals difficulty and lifting/carrying difficulty—that demonstrated robust predictive power. The extreme gradient boosting (XGBoost) algorithm exhibited superior performance across training (AUC 0.963, 95% CI: 0.951–0.975), internal validation (AUC 0.940, 95% CI: 0.924–0.956), and external validation (AUC 0.850, 95% CI: 0.832–0.868) datasets. This model significantly outperformed traditional frailty indices in predicting CKD progression (AUC 0.916 vs. 0.701, *p* < 0.001), cardiovascular events (AUC 0.789 vs. 0.708, *p* < 0.001), and mortality (time-dependent AUC 0.767 − 0.702 vs. 0.690 − 0.627, *p* < 0.001). SHAP analysis provided transparent insights into model predictions, facilitating clinical interpretation.

**Conclusion:**

Our simplified frailty assessment tool demonstrates robust performance across multiple health outcomes while minimizing measurement burden. The model’s superior predictive capabilities for CKD progression, cardiovascular events, and mortality underscore its potential utility for risk stratification.

**Supplementary Information:**

The online version contains supplementary material available at 10.1186/s12967-025-06728-4.

## Introduction

Frailty, characterized as a state of increased vulnerability resulting from age-associated decline across multiple physiological systems, has emerged as a critical determinant of health outcomes in aging populations [1, 2]. This syndrome represents more than the sum of comorbidities; rather, it reflects a fundamental biological vulnerability that predisposes individuals to disproportionate health deterioration when faced with stressors [3]. Two predominant conceptual approaches to frailty have evolved: the phenotype model, as known as frailty phenotype, which conceptualizes frailty as a specific syndrome with diagnostic criteria including unintentional weight loss, exhaustion, decreased muscle strength, slow gait speed, and reduced physical activity [4]; and the cumulative deficit model, the frailty index, which views frailty as the accumulation of deficits across multiple health domains [5, 6].

Despite its demonstrable clinical significance, frailty assessment remains inadequately implemented in routine clinical practice. Primary care physicians seldom utilize standardized frailty assessment tools in routine practice. The translation gap between research evidence and clinical application persists largely due to three key challenges: complexity of assessment protocols, time constraints in clinical settings, and limited resources for specialized testinge [7, 8]. This challenge is particularly pronounced in chronic kidney disease (CKD) populations, who already face complex medical management and would benefit significantly from practical frailty assessment methods.

Efforts to enhance the clinical applicability of frailty assessment have stimulated the development of streamlined assessment instruments, such as Rockwood’s Clinical Frailty Scale [9] and Howlett’s laboratory-based frailty index [10]. However, these approaches either sacrifice assessment precision or maintain substantial measurement burden. The Clinical Frailty Scale may have limited applicability across diverse populations due to its inherent subjectivity, which compromises its reliability for cross-cultural and cross-study comparisons [11–13]. Recent advances in machine learning offer opportunities for developing efficient predictive tools, particularly in identifying key variables and their complex interactions [14]. However, most machine learning applications in frailty assessment primarily focus on maximizing predictive performance rather than clinical practicality, typically incorporating numerous variables or requiring specialized measurements [15].

An ideal frailty assessment tool should balance predictive accuracy with clinical feasibility, utilizing readily available information without requiring additional specialized testing [16]. Such a tool could efficiently identify high-risk individuals during routine clinical care, facilitating early intervention and more effective allocation of healthcare resources [17]. Furthermore, transparent model interpretation mechanisms would enhance clinician trust and willingness to apply the tool [18]. Currently, there is a critical gap in the availability of simplified frailty assessment tools that maintain high predictive performance while ensuring clinical feasibility, particularly for CKD patients.

In this study, we developed and validated a machine learning-based simplified frailty diagnostic model utilizing four independent cohorts (NHANES, CHARLS, CHNS, and SYSU3 CKD) for multi-level validation. We systematically screened key predictive variables and compared 12 machine learning algorithms to construct an efficient yet simplified frailty prediction model. We further evaluated the model’s performance in predicting CKD progression, cardiovascular events, and all-cause mortality.

## Method

### Study design and population

**Fig. 1:**
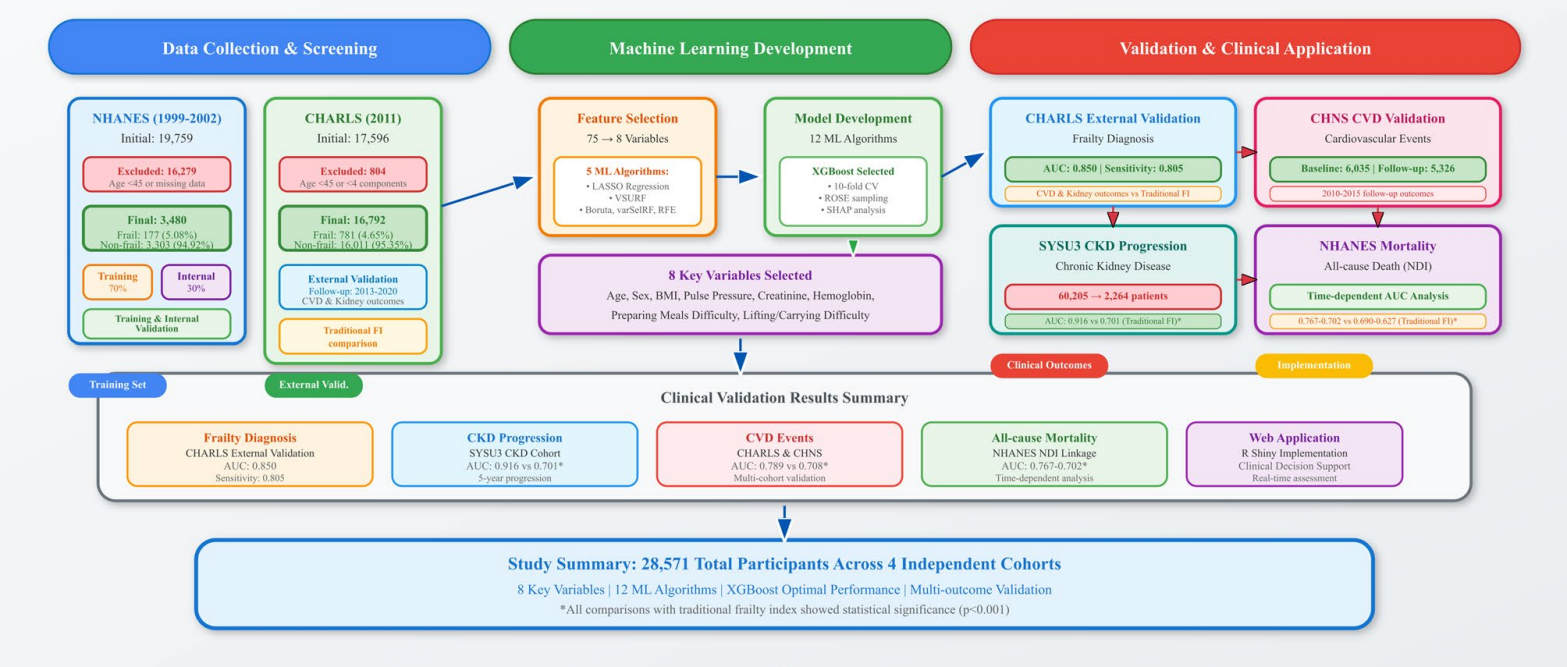
Comprehensive Methodology Framework for Machine Learning-Based Frailty Assessment Tool Development

We conducted a multi-cohort study using four independent for model development and validation. The research framework encompassed participant selection, variable identification, model development, and clinical validation across multiple outcomes (Fig. 1).

The NHANES cohort (1999–2002) served as our primary training and internal validation dataset. From 19,759 initial participants, after applying inclusion criteria (age ≥ 45 years and allowing maximum one missing modified Fried phenotype components), 3,480 participants were included in the final analysis (177 frail [5.08%], 3,303 non-frail [94.92%]).

The CHARLS cohort provided external validation with 2011 baseline data, with frailty assessment also through a modified Fried phenotype. From 17,596 initial participants, after screening for individuals with at least 4 modified Fried phenotype components and age ≥ 45 years, 16,792 individuals were included (781 frail, 16,011 non-frail; frailty prevalence 4.65%). This cohort also provided longitudinal data (2013–2020) for cardiovascular events and kidney function decline outcomes.

The CHNS cohort, without relevant variables for frailty phenotype assessment, contributed to clinical validation for cardiovascular outcomes. At 2009 baseline, 6,035 subjects had complete data of predictive factors after feature screening in the training set. For cardiovascular event outcomes (2010–2015), 5,326 subjects with follow-up data were analyzed.

The SYSU3 CKD cohort consisted of non-dialysis CKD patients from our institution (2011–2019). From 60,205 patients meeting initial CKD diagnostic criteria, 2,264 patients with complete predictive model data were included after feature screening.

### Frailty assessment and variable selection

Frailty diagnosis in our study was based on the modified Fried frailty phenotype [4], which consists of five key components as defined in Table S1: (1) Shrinking (unintentional weight loss of ≥ 5 kg in the previous year or BMI ≤ 18.5 kg/m²); (2) Slowness (reduced gait speed with sex and height-specific cutoffs); (3) Low physical activity (weekly energy expenditure below sex-specific thresholds calculated using metabolic equivalent of task values or self-reported activity level as “less active” compared to peers); (4) Weakness (difficulty lifting or carrying weights over 5 kg, typically assessed using grip strength with BMI and sex-specific cutoffs); and (5) Exhaustion (positive responses to specific items from the Center for Epidemiologic Studies Depression Scale regarding effort and motivation).Individuals with ≥ 3 positive components were categorized as frail, while those with 0–2 positive components were classified as non-frail.

We selected the modified Fried frailty phenotype over other assessment methods for several methodological considerations. Unlike subjective assessment tools such as the FRAIL questionnaire [19] and Clinical Frailty Scale [20], which rely on clinical judgment, the Fried phenotype utilizes objective, quantifiable measurements including gait speed assessment and calculated physical activity levels. This objectivity reduces inter-rater variability and enhances reproducibility across diverse clinical settings. Additionally, the Fried phenotype has been extensively validated within the NHANES database [21], providing methodological consistency for our primary training dataset and enabling direct comparison with established literature.

To ensure clinical applicability, we initially extracted 75 potential predictors from the NHANES dataset based on established frailty index [6, 22], covering multiple health domains as listed in Table S2. We employed a screening strategy combining unsupervised and supervised methods to identify the most predictive variables. During data preprocessing, we applied a 20% missing threshold through missing value analysis and eliminated near-zero variance variables and features with high multicollinearity (correlation coefficient > 0.7). Afterwards, a supervised feature selection approach was applied using five complementary algorithms: Least Absolute Shrinkage and Selection Operator (LASSO) regression with cross-validation; Variable Selection Using Random Forests (VSURF); Boruta algorithm; Variable Selection via Random Forest (varSelRF); and Recursive Feature Elimination (RFE). Finally, we conducted intersection analysis to assess consistency across algorithms and determine the core feature set. This approach resulted in the identification of 8 core features consistently selected by all five algorithms: age, sex, body mass index (BMI), pulse pressure, creatinine, hemoglobin, and preparing meals difficulty and lifting/carrying difficulty.

### Dataset selection and validation strategy

Our multi-cohort design was based on data availability and validation requirements. NHANES was selected for training due to its comprehensive variable collection (75 potential predictors) enabling systematic feature selection, and its multi-ethnic, nationally representative population providing robust model development. Only NHANES and CHARLS contained sufficient data for complete frailty phenotype assessment using modified Fried criteria, while CHNS lacked frailty diagnostic variables (containing only cardiovascular outcome data) and SYSU3 CKD focused specifically on kidney disease progression outcomes. This strategy enhances model generalizability across diverse populations and healthcare systems, following established validation practices for clinical prediction models.

### Model development and evaluation

We further constructed and validated frailty diagnostic models using the variables identified. To determine the optimal modeling approach, we systematically evaluated 12 machine learning algorithms across four categories: (1) Ensemble learning models: Extreme Gradient Boosting (XGBoost), Random Forest (RF), C5.0 decision trees, Adaptive Boosting (AdaBoost), and Gradient Boosting Machine (GBM); (2) Neural network models: Neural Network (NN) and Multi-layer Perceptron (MLP); (3) Distance and boundary-based models: Support Vector Machine (SVM) and K-Nearest Neighbors (KNN); and (4) Probabilistic models: Logistic Regression (LR), Naive Bayes (NB), and Gaussian Process (GP). The NHANES dataset was randomly divided into training (70%) and internal validation (30%) sets, stratified by frailty status. To address class imbalance, we applied Random Over-Sampling Example (ROSE) techniques during the training phase. Models were trained using 10-fold cross-validation, with hyperparameters systematically optimized through grid search methods to maximize performance while preventing overfitting.

Model performance was evaluated using multiple metrics: sensitivity, specificity, accuracy, area under the receiver operating characteristic curve (AUC), and root mean square error (RMSE).

We also developed a comprehensive scoring system to balance predictive accuracy with clinical applicability. This scoring system incorporated the following components.Overall score: A weighted composite metric reflecting model performance across all datasets, with differential weighting for external validation (0.6), internal validation (0.3), and training (0.1) to prioritize generalizability to new populations. Accuracy drop: The absolute difference in accuracy between training and external validation datasets, with smaller values indicating better resistance to overfitting and stronger generalization capability. Sensitivity drop: The absolute difference in sensitivity between training and external validation datasets, quantifying the model’s consistency in correctly identifying frail individuals across different populations. Balance score: The weighted average of absolute differences between sensitivity and specificity across all three datasets, with lower scores indicating more balanced performance across positive and negative case identification. Clinical score: Focused on external validation performance, combining AUC (weighted 0.4) with balanced sensitivity and specificity measures (weighted 0.3 each) to reflect real-world clinical utility. To determine the optimal algorithm, we ranked each model on these five dimensions, resulting in Overall rank, Overfitting rank (based on accuracy and sensitivity drops), Balance rank, and Clinical rank. The Average rank was calculated as the mean of these four ranking categories, with lower values indicating superior overall performance across all evaluation criteria.

### Clinical outcomes

We evaluated the model’s clinical utility across three key health outcomes of significant relevance to aging populations. For CKD progression in the SYSU3 CKD cohort, progression was defined by serum creatinine doubling, estimated glomerular filtration rate (eGFR) decline ≥ 40%, or acute kidney injury requiring hospitalization, with primary focus on whether CKD progression occurred within 5 years of follow-up. In the CHARLS cohort, rapid kidney function decline was defined as eGFR reduction exceeding 16 ml/min/1.73 m² between two follow-up periods (2011–2015), representing an annual decline > 4 mL/min/1.73 m² [23].

For cardiovascular disease (CVD) prediction, we assessed the model’s performance in predicting new cardiovascular events in both the CHARLS and CHNS cohorts. In CHARLS, cardiovascular event was systematically collected through questionnaire item DA007 across five survey waves (2011–2020), documenting doctor-diagnosed heart attacks, coronary heart disease, angina, heart failure, and strokes. In CHNS, events were identified through structured questionnaires recording myocardial infarction and stroke diagnoses through questionnaire item U24J, U24J2, U24L and U24V.

For all-cause mortality, we further validated the model with traditional frailty index for predicting all-cause mortality in the NHANES cohort, with death information obtained through National Death Index (NDI) linkage.

### Model interpretability and clinical implementation

To enhance model transparency and facilitate clinical adoption, we applied Shapley Additive exPlanations (SHAP) to analyze the XGBoost model. SHAP values quantify each feature’s contribution to predictions while providing measures of variable importance and effect direction. We visualized global feature importance using the shapviz package in R. Based on these results, we developed an interactive web-based tool using R Shiny, allowing healthcare providers to input the 8 key parameters and receive immediate risk assessment results.

### Statistical analysis

We performed all statistical analyses using R (version 4.1.0). Continuous variables were presented as mean ± standard deviation or median (interquartile range) based on their distribution, while categorical variables were expressed as counts and percentages. Between-group comparisons were conducted using Student’s t-test or Wilcoxon rank-sum test for continuous variables and chi-square test or Fisher’s exact test for categorical variables as appropriate.Model performance was assessed through discrimination metrics including sensitivity, specificity, accuracy, and area under the receiver operating characteristic curve (AUC). We constructed 95% confidence intervals for these metrics using the DeLong method. For survival analysis, we used Cox proportional hazards models and calculated time-dependent AUC. All analyses were performed using R version 4.1.0 with relevant packages, including caret, xgboost, randomForest, glmnet, VSURF, Boruta, varSelRF, e1071, nnet, and SHAP. Statistical significance was set at *P* < 0.05 for all analyses.

This study adheres to the Transparent Reporting of a multivariable prediction model for Individual Prognosis or Diagnosis (TRIPOD) statement for the development and validation of prediction models. A completed TRIPOD checklist is provided in Supplementary Table S6.

## Results

### Feature selection using machine learning algorithms

**Fig. 2:**
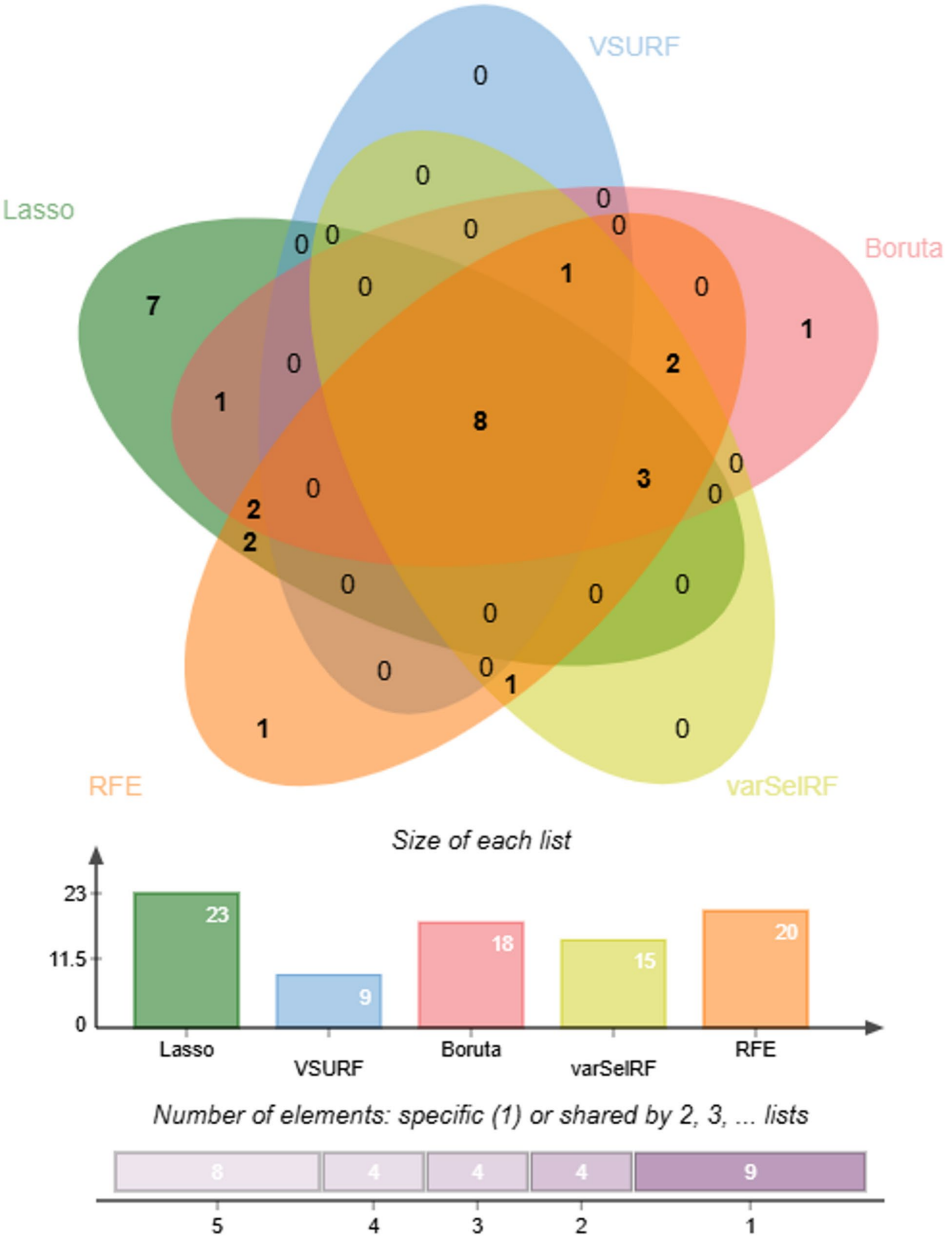
Intersection Analysis of Features Selected by Five Machine Learning Algorithms

In unsupervised feature selection, we reduced the initial 75 variables to 60. For supervised feature selection, we employed five complementary, each yielding different candidate variable sets as shown in Fig. 2. The LASSO algorithm identified 23 important variables, representing the largest initial feature set among all methods. The VSURF approach was the most stringent, selecting only 9 relevant features. The Boruta algorithm identified 18 statistically significant variables by comparing real features with artificially created “shadow variables.” The varSelRF method retained 15 variables after iterative elimination of less important predictors, while RFE selected 20 features that collectively optimized classification accuracy. The intersection analysis revealed that 8 core features were identified by all five algorithms: age, sex, BMI, pulse pressure, creatinine, hemoglobin, preparing meals difficulty, and lifting/carrying difficulty.

### Comparison of predict model variables across four cohorts

Our study included 19,938 participants across four independent cohorts (Table 1), with significant demographic and clinical differences between cohorts (all variables *P* < 0.001). The SYSU3 CKD cohort had the highest mean age (69.49 ± 13.95 years), while the CHARLS cohort had the lowest mean age (59.81 ± 9.22 years). BMI and pulse pressure followed similar patterns. Creatinine levels were significantly higher in the SYSU3 CKD cohort (196.53 ± 188.94 µmol/L) compared to other cohorts. Hemoglobin levels were relatively similar, ranging from 13.64 to 14.36 g/dL. The CHARLS and CHNS cohorts performed best in meal preparation (96.18% and 95.72% without difficulty, respectively), while the SYSU3 CKD cohort performed worst (only 66.08% without difficulty), with a much higher proportion reporting severe difficulty (13.41%). For lifting/carrying heavy objects, 93.63% in the CHARLS cohort reported no difficulty, compared to only 47.70% in the SYSU3 CKD cohort, with 25.68% reporting severe difficulty.

**Table 1:** Comparison of predictive variables across four datasets

Variables	NHANES	CHARLS	CHNS	SYSU3 CKD	*P*-value
Age (years)	66.63 (13.01)	59.81 (9.22)	68.77 (6.96)	69.49 (13.95)	< 0.001
Sex					< 0.001
-Male	1723 (49.51%)	4218 (46.76%)	2483 (46.62%)	1443 (68.36%)	< 0.001
-Female	1757 (50.49%)	4803 (53.24%)	2843 (53.38%)	688 (31.64%)	
BMI (kg/m²)	28.55 (5.98)	23.48 (3.87)	23.08 (3.49)	30.24 (7.34)	< 0.001
Pulse Pressure (mmHg)	66.53 (24.42)	55.15 (15.37)	50.79 (12.44)	70.86 (25.14)	< 0.001
Creatinine (µmol/L)	83.15 (64.21)	69.31 (21.07)	91.98(20.80)	196.53 (188.94)	< 0.001
Hemoglobin (g/dL)	14.22 (1.43)	14.36 (2.21)	13.81 (2.02)	13.64 (1.80)	< 0.001
Difficulty Preparing Meals					
- None	3080 (88.51%)	8676 (96.18%)	5098 (95.72%)	1395 (66.08%)	< 0.001
- Low	234 (6.72%)	0 (0.00%)	132 (2.48%)	272 (12.89%)	
- Medium	80 (2.30%)	0 (0.00%)	26 (0.49%)	161 (7.63%)	
- High	86 (2.47%)	345 (3.82%)	70 (1.31%)	283 (13.41%)	
Difficulty Lifting/Carrying					< 0.001
- None	2474 (71.09%)	8446 (93.63%)	4343 (81.54%)	1007 (47.70%)	
- Low	561 (16.12%)	0 (0.00%)	638 (11.98%)	438 (20.75%)	
- Medium	201 (5.78%)	0 (0.00%)	124 (2.33%)	124 (5.87%)	
- High	244 (7.01%)	575 (6.37%)	221 (4.15%)	542 (25.68%)	

After dividing the NHANES cohort into training and internal validation dataset, all predictive variables showed no significant differences between sets (all *P* > 0.05), indicating appropriate randomization. Age (66.63 ± 13.15 vs. 66.62 ± 12.44 years, *P* = 0.977), sex ratio (approximately 49% male, *P* = 0.792), body mass index (28.65 ± 6.06 vs. 28.39 ± 5.82 kg/m², *P* = 0.304), pulse pressure (67.08 ± 24.72 vs. 65.59 ± 25.11 mmHg, *P* = 0.158), creatinine (83.71 ± 65.36 vs. 85.00 ± 75.34 µmol/L, *P* = 0.65), and hemoglobin (14.21 ± 1.45 vs. 14.24 ± 1.42 g/dL, *P* = 0.662) all showed no statistical differences(Table S3).

### Model construction and selection

Table 2 shows the parameters of these algorithms across training, internal validation, and external validation datasets. While all models performed well in training (AUC range: 0.934-1.000), their performance varied considerably in validation cohorts. Table 2 and Table S4 shows that the AdaBoost algorithm achieved perfect performance metrics in the training set (sensitivity, specificity, accuracy, and AUC all 1.000, RMSE 0.166), indicating its high fitting ability for training data. However, this performance was not fully reproduced in validation datasets, suggesting potential overfitting. In the internal validation set, AdaBoost maintained relatively high performance (sensitivity 0.826, specificity 0.914, accuracy 0.830), but sensitivity significantly decreased to 0.471 in the external validation set. Random Forest also performed excellently in the training set (AUC 0.997, RMSE 0.218) but similarly showed performance decline in the external validation set, with sensitivity dropping from 0.856 to 0.595.

**Table 2:** Comparison of model performance metrics in different dataset

Model	Training Set			Internal Validation Set			External Validation Set		
	Sens	Spec	AUC	Sens	Spec	AUC	Sens	Spec	AUC
XGBoost	0.821	0.990	0.963	0.803	0.914	0.940	**0.805**	0.738	0.850
Random Forest	0.856	**0.999**	**0.997**	0.776	0.914	0.932	0.595	0.834	0.850
C5.0	0.853	0.932	0.954	0.815	0.886	0.934	0.477	0.897	0.845
AdaBoost	**1.000**	**1.000**	**1.000**	**0.826**	0.914	0.914	0.471	0.896	0.830
GBM	0.861	0.984	0.981	0.809	0.886	0.935	0.542	0.866	0.854
Neural Network	0.840	0.974	0.959	0.805	0.886	0.934	0.468	0.901	0.838
MLP	0.845	0.980	0.953	0.797	0.886	0.928	0.532	0.866	0.839
SVM	0.773	**0.999**	0.949	0.747	**1.000**	**0.943**	0.605	0.831	0.852
KNN	0.844	0.982	0.967	0.811	0.914	0.939	0.525	0.881	0.847
Logistic Regression	0.812	0.989	0.951	0.771	0.914	0.934	0.588	0.831	0.839
Naive Bayes	0.855	0.79	0.934	0.842	0.829	0.929	**0.939**	0.671	**0.857**
Gaussian Process	0.822	0.986	0.956	0.789	0.914	0.937	0.538	0.854	0.842

Note: Sen = Sensitivity; Spec = Specificity; AUC = Area Under the Receiver Operating Characteristic Curve. Accuracy and RMSE values are available in Supplementary Table S4. Best performance for each metric within each dataset is highlighted in bold

In contrast, the XGBoost model demonstrated more stable performance across all three datasets, with sensitivity of 0.821, 0.803, and 0.805 in the training, internal validation, and external validation sets, respectively, indicating better generalization capability. In the external validation set, the Naive Bayes algorithm showed the highest sensitivity (0.939), but its specificity (0.671) and accuracy (0.685) were the lowest. SVM and KNN had excellent AUC performance in the internal validation set (0.943 and 0.939, respectively), but their performance relatively declined in the external validation set. KNN’s RMSE in the external validation set reached 0.400, the highest among all models, indicating larger deviations between predicted and actual values.

Overall, while all models performed well on training data, their generalization ability when facing new data varied significantly. XGBoost, SVM, and Naive Bayes showed stronger generalization capability in the external validation set, with AUCs of 0.850, 0.852, and 0.857, respectively. As shown in Table S5, XGBoost achieved the highest total score (0.840) and lowest average ranking (3.75) in terms of accuracy, sensitivity, balance, and clinical utility metrics.

**Fig. 3:**
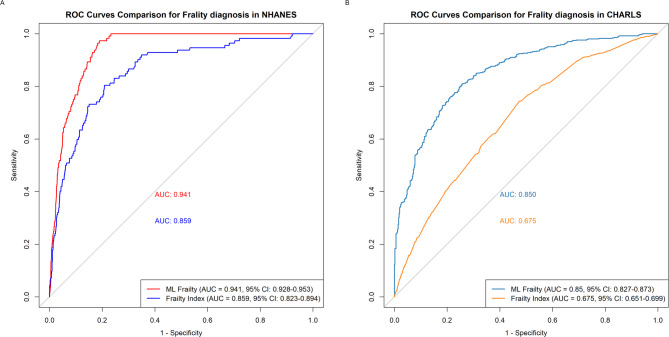
Comparison of ROC Curves Between Machine Learning-Based Frailty Diagnostic Model and Traditional Frailty Index Across Different Cohorts. (**A**) NHANES cohort and (**B**) CHARLS external validation cohort

Our machine learning frailty diagnostic model based on the XGBoost algorithm outperformed traditional frailty index in both NHANES and CHARLS datasets (Fig. 3), showing superior discrimination (AUC: 0.941, 95% CI: 0.928–0.953) compared to traditional cumulative frailty index (AUC: 0.859, 95% CI: 0.823–0.894).

### Clinical application of the model

**Fig. 4:**
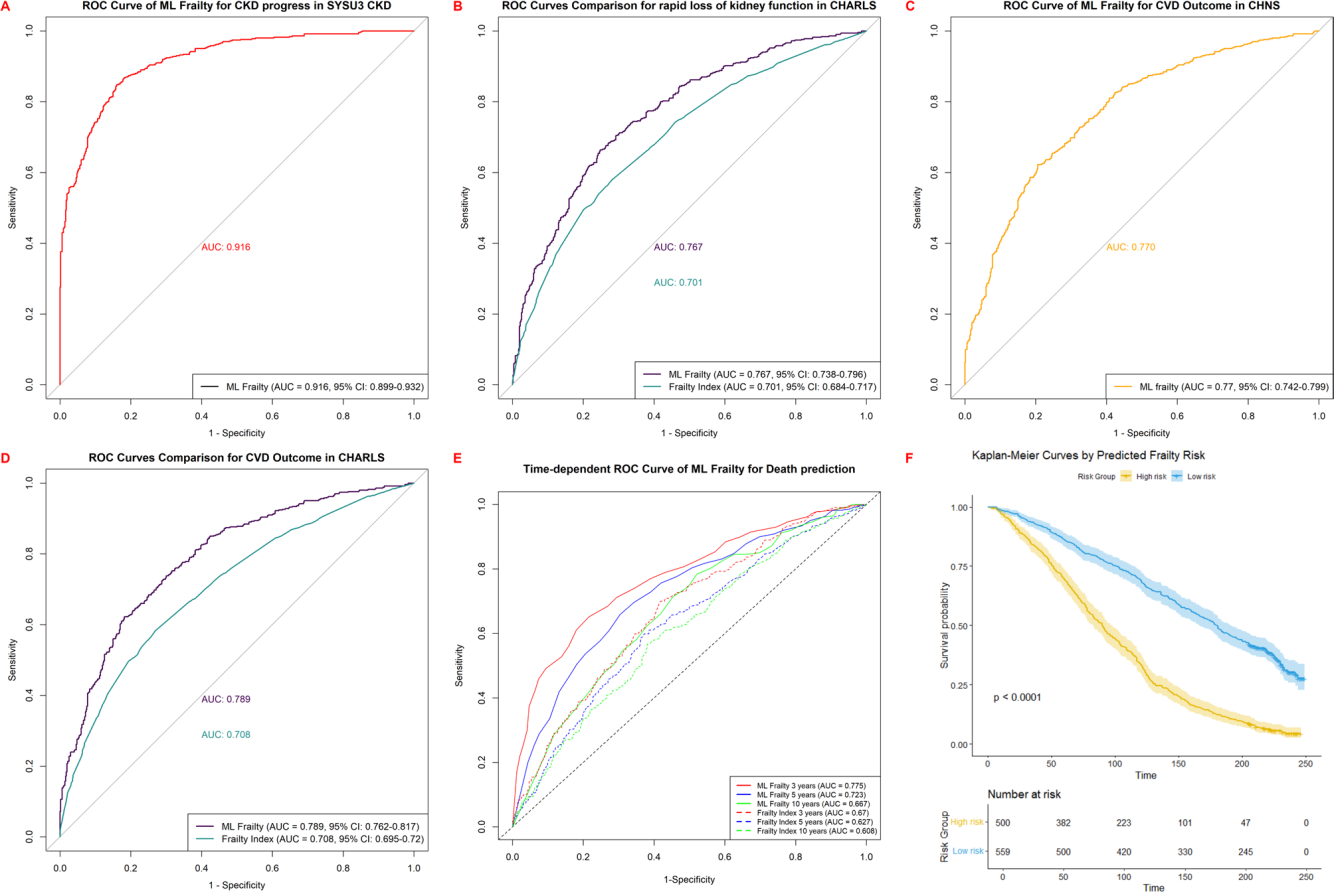
Clinical Utility of the Machine Learning Frailty Model for Predicting Multiple Health Outcomes Across Diverse Cohorts. (**A**) CKD progression prediction in SYSU3 CKD cohort; (**B**) Comparison with traditional frailty index for rapid kidney function decline prediction in CHARLS; (**C**) Cardiovascular event prediction in CHNS; (**D**) Comparison with traditional frailty index for cardiovascular outcomes in CHARLS; (**E**) Time-dependent mortality prediction in NHANES compared with traditional frailty metrics; (**F**) Kaplan-Meier survival curves stratified by predicted frailty risk categories

The machine learning frailty diagnostic model demonstrated robust clinical utility across various health outcomes. For CKD progression prediction in SYSU3 CKD cohort (Fig. 4A), our model achieved high discrimination (AUC: 0.916, 95% CI: 0.899–0.932). In the CHARLS cohort (Fig. 4B), the model significantly outperformed traditional frailty index in predicting rapid kidney function decline (ML frailty diagnostic model AUC: 0.767, 95% CI: 0.758–0.786 vs. frailty index AUC: 0.701, 95% CI: 0.684–0.717).

For cardiovascular event prediction in cohort (Fig. 4C), the model also performed well (AUC: 0.77, 95% CI: 0.742–0.799). More importantly, in the CHARLS cohort (Fig. 4D), it demonstrated superior predictive capability for CVD compared to frailty index (ML frailty diagnostic model AUC: 0.789, 95% CI: 0.762–0.817 vs. traditional frailty index AUC: 0.708, 95% CI: 0.695–0.720).

For mortality prediction in NHANES (Fig. 4E), the AUC at different follow-up time points ranged from 0.767 at 36 months to 0.702 at 120 months, with AUC values ranging from 0.767 (95% CI: 0.752–0.783) at 36 months to 0.702 (95% CI: 0.689–0.715) at 120 months of follow-up, consistently outperforming the frailty index (AUC range: 0.627–0.690 across time points, *p* < 0.001 for all comparisons).Kaplan-Meier survival curves (Fig. 4F), further confirmed that survival probability differences between groups stratified by predicted risk according to the machine learning frailty diagnostic model were significant (log-rank *P* < 0.001), with the high-risk group showing substantially higher mortality during follow-up.

### Model interpretability and clinical interactive application

**Fig. 5:**
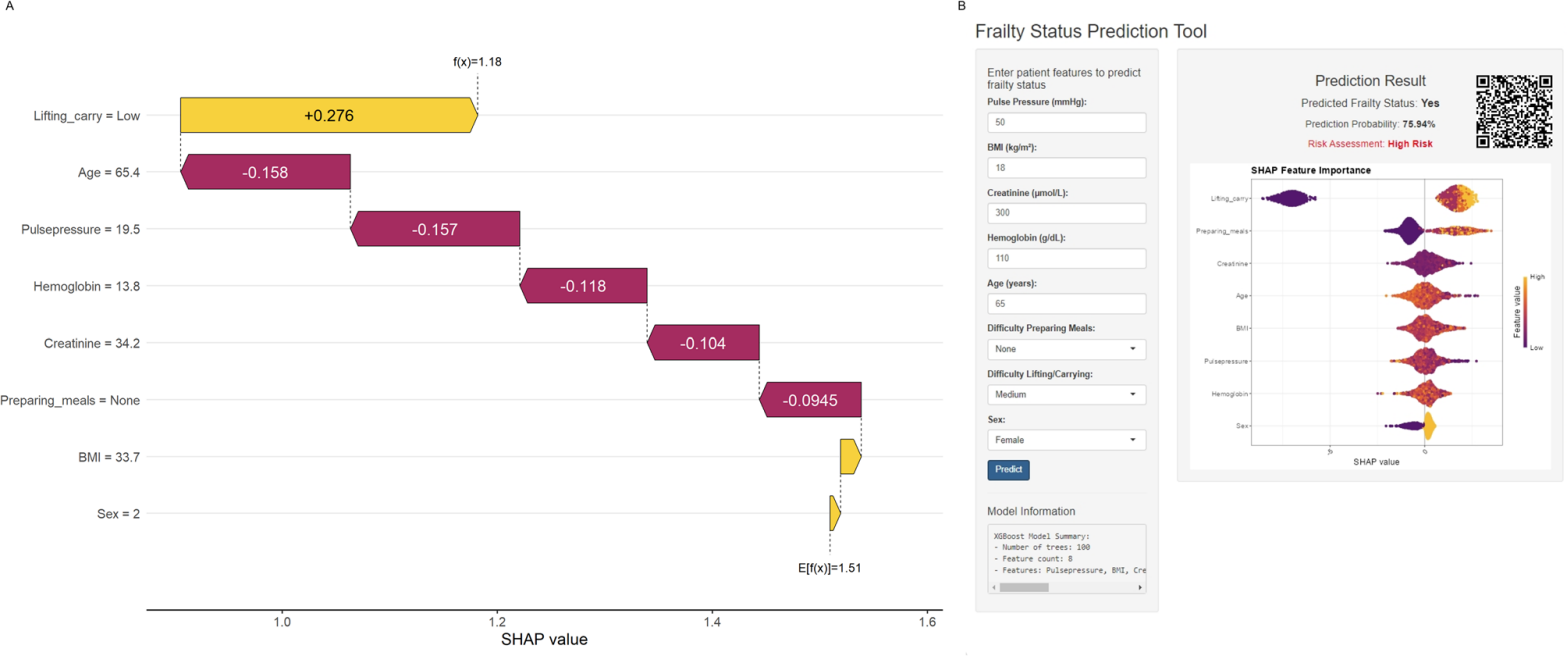
SHAP Analysis and Clinical Web Application of the Machine Learning Frailty Model. (**A**) SHAP Values of Feature Variables and Their Impact on Prediction Results (**B**) Web Application Interface for clinical implementation

SHAP (SHapley Additive exPlanations) value analysis of model feature importance (Fig. 5) revealed that functional capability variables were the strongest predictors of frailty risk. Difficulty lifting/carrying had the highest positive SHAP value (+ 0.276 Laboratory and physiological parameters exhibited significant negative correlation with frailty, with hemoglobin exhibiting the strongest negative impact (-0.118), followed by creatinine (-0.104), pulse pressure (-0.157), and age (-0.158). Difficulty preparing meals, BMI, and sex showed smaller but significant contributions to model predictions.

We developed an interactive web-based application using R Shiny (Fig. 5). The platform features a straightforward interface for healthcare providers to input the eight predictive parameters. After data entry, users simply click the “Predict” button to generate comprehensive assessment results. These results are presented in three complementary forms: clear frailty status determination, probability score, and risk stratification category.

A QR code in the bottom right corner of the system provides quick access for healthcare personnel to access this tool via mobile devices, enhancing application flexibility in clinical practice. The application has been deployed online (https://ckdpredictionmodel.shinyapps.io/CKD-Frailty-Model/), freely available to healthcare professionals for incorporation into clinical assessment workflows.

## Discussion

Our study developed and validated a machine learning-based simplified frailty prediction model by systematically applying multiple feature selection algorithms to identify 8 key variables from numerous potential predictors, constructing a both streamlined and efficient frailty assessment tool. The model demonstrated robust predictive performance across multiple cohorts and different health outcomes, providing a practical solution for clinical frailty assessment.

Our machine learning frailty diagnostic model achieves remarkable predictive performance using only 8 readily available clinical parameters. Traditional Fried frailty phenotype requires specialized physical tests such as gait speed measurement, grip strength evaluation, and detailed physical activity quantification [4], which present significant implementation barriers in routine clinical practice. These complex measurements represent major barriers to widespread application of frailty assessment [1, 24, 25]. Compared to traditional cumulative frailty index requiring 30–70 variables [5, 22, 26], and simplified versions needing 20–30 items [27], our model substantially reduces assessment complexity. The Clinical Frailty Scale (CFS), a 9-level assessment tool based on clinical judgment, has been confirmed by Church et al.‘s meta-analysis. However, as Rockwood et al. note, CFS heavily relies on subjective judgment by assessors, potentially leading to scoring variability [20]. Similarly, the FRAIL scale, a screening tool comprising 5 simple questions, showed limited predictive power (AUC = 0.6) for mortality in community-dwelling older adults in Woo et al.‘s study [28], significantly lower than our model’s performance in the NHANES cohort (AUC = 0.702–0.767).

Our approach directly addresses the primary barriers to widespread implementation of frailty assessment in routine clinical practice– namely, time constraints and resource limitations facing healthcare providers. When comparing our model with contemporaneous machine learning frailty diagnostic approaches, several distinguishing advantages emerge in relation to variable selection strategies, model performance, and clinical feasibility. In the English Longitudinal Study of Ageing cohort, investigators identified age, chair-rise test, household wealth, balance problems, and self-rated health as key frailty predictors using Random Forest models (AUC 0.92) [29]. However, their approach required comprehensive functional assessments including mobility evaluations, balance testing, and socioeconomic questionnaires.

Tarekegn et al.‘s learning model based on UK Biobank data, while highly accurate (AUC = 0.81), is limited by its “black box” nature, restricting clinical interpretability [30]. Meanwhile, our model demonstrates clear advantages over Kim et al.‘s 16-variable model (AUC = 0.77) [31], through significant variable simplification while maintaining or improving predictive capability. The electronic frailty index (eFI) developed by Clegg et al. [16] based on routine primary care data showed satisfactory performance for predicting hospitalization and mortality in the original validation study. Recent innovations have explored wearable sensor data [32] and vocal biomarkers [33] for frailty detection, showing promise but facing implementation challenges. These emerging technologies require specialized equipment and technical expertise, and critically, have not undergone large-scale population validation across diverse demographic groups, limiting their real-world applicability.By comparison, our 8-variable model achieved similar or superior predictive performance while substantially reducing required data points, significantly lowering implementation complexity. Anyway, when comparing our machine learning frailty diagnostic model with published frailty assessment tools, we must cautiously interpret performance metrics, as different studies use different datasets, evaluation criteria, and validation methods [34].

Our approach prioritizes variables that are easily accessible in electronic health records or assessable through brief self-reported questions, facilitating seamless integration with standard clinical workflows. This simplified approach addresses key challenges in clinical frailty screening. As other researchers have observed, no standardized screening test for frailty or pre-frailty currently exists, and time and resource constraints are primary barriers to implementing comprehensive assessments in clinical settings [35]. Our approach provides a practical solution, substantially reducing assessment burden while maintaining predictive accuracy.

The variables in our model accurately reflect the multi-system functional impairment characteristic of frailty. Functional indicators (preparing meals difficulty and lifting/carrying difficulty) as the most important predictors, embodying the core manifestation of physical function decline central to what is often termed “functional frailty” [36]. This emphasis on functional decline is consistent with recent systematic evidence showing that frailty and sarcopenia share common metabolic, inflammatory, and hematologic abnormalities [37]. These functional indicators reflect not only sarcopenia but also the integration of balance, coordination, and cognitive function. This aligns with other research findings, such as the importance of six-minute walk tests and grip strength assessments in predicting frailty [38, 39]. Hemoglobin as a key predictor is closely related to anemia and inflammatory states associated with chronic diseases [40]. Anemia, particularly iron-deficiency anemia, has been proven to be associated with decreased muscle function, limited physical activity, and cognitive decline, all of which are key components of the frailty syndrome [41]. Kidney function is a key biomarker linking for frailty, with declining renal function independently increasing frailty risk through mechanisms such as uremic toxin accumulation, mitochondrial dysfunction, and muscle catabolism [42, 43]. The importance of pulse pressure highlights the association between vascular physiology and frailty, potentially reflecting the impact of vascular aging processes such as arterial stiffness on tissue perfusion [44]. The bidirectional influence of body mass index (both high and low increasing risk) reflects the role of weight changes (especially sarcopenia) in frailty development [45].

The XGBoost algorithm applied in our study stood out in comparison of multiple machine learning models, demonstrating excellent performance. This algorithm excels at capturing non-linear relationships and complex interactions between variables [46], which may explain its superior performance compared to traditional frailty index, which typically rely on simple summation of deficits without considering differential weights or synergistic effects. Compared to traditional statistical methods, machine learning approaches demonstrate significant advantages in handling complex, non-linear clinical problems. A key advancement of our approach is the integration of model interpretability and clinical implementation. Through SHAP value analysis, we provide transparent insights into factors driving individual risk predictions, enhancing clinician trust and facilitating targeted interventions [47]. This interpretability is crucial for clinical adoption of decision support systems. Our interactive web application transforms complex machine learning outputs into intuitive clinical tools, supporting real-time risk assessment and visualizing feature importance. This implementation strategy addresses common “black box” concerns in clinical applications of machine learning [48, 49]. Consistent with Shah et al.‘s research [50], such user-friendly interfaces may be key factors in promoting clinical adoption.

Our model’s clinical validation results emphasize its potential impact across multiple domains. Its strong performance in predicting CKD progression (SYSU3 CKD: AUC = 0.916; CHARLS: accuracy = 0.78) suggests its value for risk stratification in nephrology practice. Similarly, the model’s ability to predict cardiovascular events (AUC: 0.77–0.789) and mortality (time-dependent AUC: 0.702–0.767) indicates its potential applications in primary care, cardiology, and geriatrics. In all these outcomes, our model consistently outperformed traditional frailty index, aligning with findings emphasizing the incremental value of frailty assessment beyond traditional cardiovascular risk prediction [31].

Our study has several limitations. First, despite our multi-cohort validation approach, generalizability to populations with different frailty prevalence or risk factor distributions requires further assessment. Selection bias may also exist as participants in population-based surveys may not fully represent clinical populations, particularly those with severe frailty who might be less likely to participate in research studies. Second, while our model reduces assessment burden, some variables still require laboratory testing, which may not be universally available in all clinical settings. Second, while our model reduces assessment burden, some variables still require laboratory testing, which may not be universally available in all clinical settings. Third, the primarily cross-sectional nature of our main analysis limits assessment of the model’s performance in tracking frailty changes over time. Longitudinal validation studies are needed to evaluate its utility in monitoring intervention effects. Additionally, our study relies on established frailty phenotypes and indices as reference standards, which themselves have limitations and may not fully capture the complex physiology of frailty. Due to the multidimensional nature of frailty, additional biomarkers or functional measurements may be needed to further enhance the precision of frailty state assessment.

Future research should focus on three key areas: (1) prospective implementation studies evaluating the model’s impact on clinical decision-making and patient outcomes; (2) developing adaptive versions that can accommodate missing data in resource-limited settings; and (3) integration with electronic health record systems, enabling automated risk stratification during routine care. Additionally, exploring how the model can guide personalized intervention strategies represents an important direction for translational research. The model’s short-term and long-term predictive capabilities should also be further studied, particularly considering that frailty is a dynamic state that may change over time. Quantifying the cost-effectiveness of early frailty intervention across different clinical contexts may also provide valuable insights for implementation.

## Conclusions

In conclusion, our machine learning-based frailty prediction model achieves robust predictive performance across diverse health outcomes using a minimized set of readily available variables. By reducing measurement burden while maintaining clinical utility, this approach addresses key barriers to routine frailty assessment. The integration of model interpretability and interactive implementation tools further enhances its potential clinical impact. Our results suggest that this efficient approach can facilitate routine assessment of frailty in clinical practice, potentially improving risk assessment and intervention timing in CKD and cardiovascular management.

## Electronic Supplementary Material

Below is the link to the electronic supplementary material.

Supplementary Material 1

## Data Availability

The datasets analyzed during the current study are available as follows: NHANES data are available from the Centers for Disease Control and Prevention repository (https://www.cdc.gov/nchs/nhanes/). NHANES mortality data are available through the National Death Index (NDI) linkage. CHARLS data are available from the China Health and Retirement Longitudinal Study official website (http://charls.pku.edu.cn/). CHNS data are available from the China Health and Nutrition Survey database (https://www.cpc.unc.edu/projects/china). The SYSU3 CKD cohort data are available from the corresponding author upon reasonable request and with appropriate data use agreements.
