# Supplementary material for: Development and multi-cohort validation of a machine learning-based simplified frailty assessment tool for clinical risk prediction
Source: J Transl Med. 2025 Aug 15;23:921. doi: 10.1186/s12967-025-06728-4 (PMC12357463; doi:10.1186/s12967-025-06728-4)
Supplement: Supplementary file 1 — Supplementary Material 1 [file 12967_2025_6728_MOESM1_ESM.docx]

**Table S1: Definitions of Frailty Phenotype Components**

| **Component** | **Definition** |
| --- | --- |
| 1. Shrinking | Unintentional loss of 5 or more kg in the previous year or body mass index (BMI) ≤18.5 kg/m² |
| 2. Slowness | Measured using gait speed over a timed 20-foot (6.1 meter), with sex and height-specific cutoffs: Women: ≤0.36 m/s (height ≤151 cm) or ≤0.43 m/s (height >151 cm); Men: ≤0.45 m/s (height ≤163 cm) or ≤0.48 m/s (height >163 cm). |
| 3. Low physical activity | Defined by either: (1) Weekly energy expenditure below sex-specific thresholds (<383 kcal/week for men, <270 kcal/week for women), calculated from physical activities including walking, cycling, household tasks, and strength training using metabolic equivalent of task (MET) values; or (2) Self-reported activity level as "less active" compared to same-age peers of the same gender |
| 4. Weakness | Operationally defined as difficulty in lifting or carrying weights over 5 kg. Standard assessment typically includes grip strength measurement with BMI and sex-specific cutoffs |
| 5. Exhaustion | Response to Center for Epidemiologic Studies Depression Scale(CES-D) questions: "I felt everything I did was an effort during last week" or "I could not get going during last week" - Present if answering "Most or all of the time" or "Occasionally or a moderate amount of the time" |

**Table S2: Variables Used in the Frailty Index and Their NHANES Codes**

| **Domain** | **Frailty index included 49 deficits** | **Frailty index from common clinical and laboratory tests** | **NHANES Variable Codes** |
| --- | --- | --- | --- |
| **Cognition** | Experience confusion/memory problems | Experience confusion/memory problems | pfq056, pfq057 |
| **Functional Dependence** | Managing money difficulty | Difficulty managing money | pfq060a, pfq061a |
|  | Walking for a quarter mile difficulty | Difficulty walking between rooms on same floor | pfq060b, pfq061b |
|  | Walking up ten steps difficulty | - | pfq060c, pfq061c |
|  | Stooping, crouching, kneeling difficulty | Difficulty stooping, crouching, kneeling | pfq060d, pfq061d |
|  | Lifting or carrying difficulty | Difficulty grasping/holding small objects | pfq060e, pfq061e |
|  | House chore difficulty | - | pfq060f, pfq061f |
|  | Preparing meals difficulty | Difficulty preparing meals | pfq060g, pfq061g |
|  | Standing up from armless chair difficulty | Difficulty standing up from armless chair | pfq060i, pfq061i |
|  | Getting in and out of bed difficulty | - | pfq060j, pfq061j |
|  | Using fork, knife, drinking from cup difficulty | Difficulty using fork and knife | pfq060k, pfq061k |
|  | Dressing yourself difficulty | Difficulty dressing yourself | pfq060l, pfq061l |
|  | Standing for long periods difficulty | - | pfq060m, pfq061m |
|  | Grasp/holding small objects difficulty | Difficulty grasping/holding small objects | pfq060p, pfq061p |
|  | Attending social event difficulty | Difficulty attending social event | pfq060r, pfq061r |
|  | Leisure activity at home difficulty | Difficulty attending social event | pfq060s, pfq061s |
|  | Push or pull large objects difficulty | Difficulty pushing or pulling large objects | pfq061t |
| **Depressive Symptoms** | Have little interest in doing things | - | ciqd008, ciqd009, dpq010 |
|  | Feeling down, depressed, or hopeless | - | dpq020, ciqd001, ciqd002 |
|  | Trouble sleeping or sleeping too much | - | dpq030, ciqd025, ciqd026 |
|  | Feeling tired or having little energy | - | dpq040 |
|  | Poor appetite or overeating | - | ciqd019, ciqd022, dpq050 |
|  | Feeling bad about yourself | - | dpq060, ciqd029 |
|  | Trouble concentrating on things | - | dpq070, ciqd043 |
| **Comorbidities** | Doctor ever said you had arthritis | Arthritis | mcq160a |
|  | Ever told you had thyroid problem | Thyroid condition | mcq160i, mcd160m, mcq160m |
|  | Ever told you had chronic bronchitis | Cough regularly | mcq160k, mcq160p |
|  | Ever told you had cancer or malignancy | Cancer | mcq220 |
|  | Ever told had congestive heart failure | Heart attack | mcq160b |
|  | Ever told you had coronary heart disease | Heart disease | mcq160c |
|  | Ever told you had angina/angina pectoris | Angina/angina pectoris | mcq160d |
|  | Ever told you had heart attack | Heart attack | mcq160e |
|  | Ever told you had a stroke | Stroke | mcq160f |
|  | Ever told you had high blood pressure | High blood pressure | bpq020 |
|  | Doctor told you have diabetes | Diabetes | diq010 |
|  | Ever told you had weak/failing kidneys | Weak/failing kidneys | kiq020, kiq022 |
|  | Urine leakage bother you? | Leaked/lost control of urine | kiq040, kiq050 |
| **Healthcare Utilization** | General health condition | Self-reported health | huq010 |
|  | Health now compared with 1 year ago | Health compared to 1 year ago | huq020 |
|  | Overnight hospital patient in last year | Overnight hospital stays | huq070, hud070, huq071 |
|  | Times received healthcare over the past year | Frequency of healthcare use | huq050, huq051 |
|  | Number of prescription medicines taken | - | rxd030, rxduse, rxd295, rxdcount |
| **Anthropometrics** | BMI | BMI | Calculated from bmxht,bmxwt |
| **Blood Pressure/Pulse** | - | Blood pressure - diastolic (60-90 mmHg) | bpxdi1, bpxdi2, bpxdi3, bpxdi4 |
|  | - | Blood pressure - systolic (90-140 mmHg) | bpxsy1, bpxsy2, bpxsy3, bpxsy4 |
|  | - | Mean arterial pressure (70-105 mmHg) | Calculated from systolic and diastolic |
|  | - | Pulse pressure (30-65 mmHg) | Calculated from systolic and diastolic |
|  | - | Pulse (60-99 bpm) | bpxpls |
| **Hematological Parameters** | Hemoglobin (g/dl) | Hemoglobin (M: 13.5-18 g/dL, W: 12-16 g/dL) | lbxhgb |
|  | Lymphocyte percent (%) | Platelet count (150-450 1000 cells/uL) | lbxpltsi |
|  | - | Mean cell volume (80-96 fL) | lbxmcvsi |
|  | Red cell distribution width | Red cell distribution width (11.6-14.6%) | lbxrdw |
|  | Segmented neutrophils percent (%) | Segmented neutrophils percent (40-80%) | lbxnepct |
| **Liver Function Markers** | Red blood cell count (million cells/μl) | Protein, total (60-78 g/L) | lbxrbcsi |
|  | - | Albumin (32-45 g/L) | lbxlypct |
|  | - | Alkaline phosphatase (20-130 U/L) | lbxsapsi |
|  | - | Bilirubin, total (2-21 umol/L) | lbdstbsi |
|  | - | Lactate dehydrogenase LDH (100-190 U/L) | lbxsldsi |
| **Renal Function Parameters** | - | Blood urea nitrogen (2.9-8.2 mmol/L) | lbdsbusi |
|  | - | Creatinine (M: 60-110 umol/L, W:45-90 umol/L) | lbdscrsi |
|  | - | Bicarbonate (21-28 mmol/L) | lbxsc3si |
|  | - | Uric acid (M:240-510 umol/L, W:160-430umol/L) | lbdsuasi |
|  | - | Phosphorus (0.74-1.52 mmol/L) | lbdsphsi |
|  | - | Sodium (136-142 mmol/L) | lbxsnasi |
|  | - | Total calcium (2.3-2.74 mmol/L) | lbdscasi |
| **Cardiovascular Markers** | - | Direct HDL-Cholesterol (1.3+mmol/L) | lbdhddsi |
|  | - | Total Cholesterol (3.88-6.47 mmol/L) | lbdtcsi |
|  | - | Triglyceride (0.11-2.74 mmol/L) | lbdstrsi |
|  | - | Glucose, serum (3.9-6.1 mmol/L) | lbdsglsi |
|  | Glycohemoglobin (%) | Glycohemoglobin levels (0-5.7%) | lbxgh |
|  | - | C-reactive protein (0-1 mg/dL) | lbxcrp |
| **Trace Elements** | - | Iron, refrigerated (10.7-26.9 umol/L) | lbdsirsi |
|  | - | Folate, RBC (376-1450 nmol/L) | lbdrbfsi |
|  | - | Vitamin B12, serum (118-701 pmol/L) | lbdb12si |
|  | - | Vitamin D (12-50 ng/mL) | lbdvidms |

**Table S3 Comparison of Predictive Variables Between Training and Testing Sets in NHANES Cohort**

| **Variables** | **Training Set (n=2785)** | **Validation dataset (n=695)** | **P-value** |
| --- | --- | --- | --- |
| Age (years) | 66.63 ± 13.15 | 66.62 ± 12.44 | 0.977 |
| Sex |  |  | 0.792 |
| -Male | 1382 (49.62%) | 341 (49.07%) |  |
| -Female | 1403 (50.38%) | 354 (50.93%) |  |
| BMI (kg/m²) | 28.65 ± 6.06 | 28.39 ± 5.82 | 0.304 |
| Pulse Pressure (mmHg) | 67.08 ± 24.72 | 65.59 ± 25.11 | 0.158 |
| Creatinine (μmol/L) | 83.71 ± 65.36 | 85.00 ± 75.34 | 0.65 |
| Hemoglobin (g/dL) | 14.21 ± 1.45 | 14.24 ± 1.42 | 0.662 |
| Difficulty Preparing Meals |  |  | 0.479 |
| - None | 2474 (88.83%) | 605 (87.05%) |  |
| - Low | 182 (6.54%) | 52 (7.48%) |  |
| - Medium | 64 (2.30%) | 16 (2.30%) |  |
| - High | 65 (2.33%) | 22 (3.17%) |  |
| Difficulty Lifting/Carrying |  |  | 0.885 |
| - None | 1985 (71.28%) | 488 (70.22%) |  |
| - Low | 446 (16.01%) | 115 (16.55%) |  |
| - Medium | 162 (5.82%) | 39 (5.61%) |  |
| - High | 192 (6.89%) | 53 (7.63%) |  |
| Frailty |  |  | 0.946 |
| - Frailty | 2643 (94.90%) | 660 (94.96%) |  |
| -Non-frail | 142 (5.10%) | 35 (5.04%) |  |

**Table S4 Comparison of Model Performance Metrics in different dataset**

|  | Training Set | | Internal Validation Set | | External Validation Set | |
| --- | --- | --- | --- | --- | --- | --- |
| Model | Accuracy | RMSE | Accuracy | RMSE | Accuracy | RMSE |
| XGBoost | 0.907 | 0.265 | 0.809 | 0.338 | 0.741 | 0.31 |
| Random Forest | 0.929 | 0.218 | 0.783 | 0.365 | 0.821 | 0.349 |
| C5.0 | 0.894 | 0.291 | 0.819 | 0.315 | 0.875 | 0.302 |
| AdaBoost | 1 | 0.166 | 0.83 | 0.341 | 0.874 | 0.316 |
| GBM | 0.924 | 0.236 | 0.813 | 0.333 | 0.849 | 0.323 |
| Neural Network | 0.908 | 0.26 | 0.809 | 0.357 | 0.878 | 0.323 |
| MLP | 0.914 | 0.259 | 0.801 | 0.356 | 0.848 | 0.331 |
| SVM | 0.888 | 0.281 | 0.76 | 0.379 | 0.819 | 0.324 |
| KNN | 0.914 | 0.258 | 0.816 | 0.339 | 0.863 | 0.4 |
| Logistic Regression | 0.902 | 0.273 | 0.778 | 0.36 | 0.818 | 0.328 |
| Naive Bayes | 0.822 | 0.401 | 0.842 | 0.383 | 0.685 | 0.546 |
| Gaussian Process | 0.905 | 0.274 | 0.796 | 0.347 | 0.838 | 0.321 |

**Table S5 Comparative Performance Analysis of Machine Learning Models for Optimal Algorithm Selection**

| **Model** | **Overall score** | **Accuracy drop** | **Sensitivity drop** | **Balance score** | **Clinical score** | **Overall rank** | **Overfitting rank** | **Balance rank** | **Clinical rank** | **Average rank** |
| --- | --- | --- | --- | --- | --- | --- | --- | --- | --- | --- |
| XGBoost | 0.840 | 0.222 | 0.016 | 0.090 | 0.811 | 1 | 11 | 1 | 2 | 3.75 |
| Naive Bayes | 0.840 | 0.249 | -0.084 | 0.171 | 0.831 | 2 | 12 | 2 | 1 | 4.25 |
| SVM | 0.822 | 0.130 | 0.168 | 0.234 | 0.785 | 3 | 7 | 6 | 3 | 4.75 |
| Random Forest | 0.818 | 0.176 | 0.261 | 0.199 | 0.782 | 4 | 10 | 3 | 4 | 5.25 |
| KNN | 0.816 | 0.104 | 0.319 | 0.258 | 0.775 | 5 | 3 | 9 | 6 | 5.75 |
| GBM | 0.815 | 0.132 | 0.319 | 0.230 | 0.779 | 6 | 8 | 5 | 5 | 6.00 |
| Logistic Regression | 0.811 | 0.133 | 0.224 | 0.206 | 0.774 | 8 | 9 | 4 | 7 | 7.00 |
| MLP | 0.807 | 0.105 | 0.313 | 0.241 | 0.769 | 10 | 4 | 7 | 8 | 7.25 |
| Gaussian Process | 0.809 | 0.118 | 0.284 | 0.244 | 0.769 | 9 | 5 | 8 | 9 | 7.75 |
| C5.0 | 0.805 | 0.079 | 0.376 | 0.281 | 0.766 | 11 | 1 | 10 | 10 | 8.00 |
| AdaBoost | 0.812 | 0.126 | 0.529 | 0.281 | 0.757 | 7 | 6 | 11 | 12 | 9.00 |
| Neural Network | 0.803 | 0.081 | 0.372 | 0.298 | 0.761 | 12 | 2 | 12 | 11 | 9.25 |

**Table S6 TRIPOD Checklist: Development and Multi-cohort Validation of a Machine Learning-Based Simplified Frailty Assessment Tool for Clinical Risk Prediction**

| **Section/Topic** | **Item** | **Checklist Item** | **Page** |
| --- | --- | --- | --- |
| **Title and Abstract** |  |  |  |
| Title | 1 | Identify the study as developing and/or validating a multivariable prediction model, the target population, and the outcome to be predicted. | 1 |
| Abstract | 2 | Provide a summary of objectives, study design, setting, participants, sample size, predictors, outcome, statistical analysis, results, and conclusions. | 1-2 |
| **Introduction** |  |  |  |
| Background and objectives | 3a | Explain the medical context (including whether diagnostic or prognostic) and rationale for developing or validating the multivariable prediction model, including references to existing models. | 3-5 |
|  | 3b | Specify the objectives, including whether the study describes the development or validation of the model or both. | 5 |
| **Methods** |  |  |  |
| Source of data | 4a | Describe the study design or source of data (e.g., randomized trial, cohort, or registry data), separately for the development and validation data sets, if applicable. | 5-6 |
|  | 4b | Specify the key study dates, including start of accrual; end of accrual; and, if applicable, end of follow-up. | 6 |
| Participants | 5a | Specify key elements of the study setting (e.g., primary care, secondary care, number of centres) and describe eligibility criteria for participants. | 6 |
|  | 5b | Give details of treatments received, if relevant. | N/A |
|  | 5c | Provide details of the follow-up, if any (e.g., length, type, assessment of outcome, loss to follow-up). | 10-11 |
| Outcome | 6a | Clearly define the outcome that is predicted by the prediction model, including how and when assessed. | 7 |
|  | 6b | Report any actions to blind assessment of the outcome to be predicted. | N/A |
| Predictors | 7a | Clearly define all predictors used in developing or validating the multivariable prediction model, including how and when they were measured. | 7-8 |
|  | 7b | Report any actions to blind assessment of predictors for the outcome and other predictors. | N/A |
| Sample size | 8 | Explain how the study size was arrived at. | 6 |
| Missing data | 9 | Describe how missing predictor data were handled (e.g., complete case analysis, single or multiple imputation). | 8 |
| Statistical analysis methods | 10a | Describe how predictors were handled in the analyses. | 8-9 |
|  | 10b | Specify type of model, all model-building procedures (including any predictor selection), and method for internal validation. | 8-9 |
|  | 10c | For validation, describe how the predictions were calculated. | 9 |
|  | 10d | Specify all measures used to assess model performance and, if relevant, to compare multiple models. | 9 |
|  | 10e | Describe any model updating (e.g., recalibration) arising from the validation, if done. | N/A |
| Risk groups | 11 | Provide details on how risk groups were created, if done. | N/A |
| Development vs. validation | 12 | For validation, identify any differences from the development data in setting, eligibility criteria, outcome, and predictors. | 6-7 |
| **Results** |  |  |  |
| Participants | 13a | Describe the flow of participants through the study, including the number of participants with and without the outcome to be predicted in the development and validation data sets. | 11-12 |
|  | 13b | Describe the characteristics of the participants (basic demographics, clinical features, available predictors), including the number with missing data for predictors and outcome. | 12-13 |
|  | 13c | For validation, show a comparison with the development data of the distribution of important variables (demographics, predictors and outcome). | 12-13 |
| Model development | 14a | Specify the number of participants and outcome events in each analysis. | 11-12 |
|  | 14b | If done, report the unadjusted association between each candidate predictor and outcome. | N/A |
| Model specification | 15a | Present the full prediction model to allow predictions for individuals (i.e., all regression coefficients, and model intercept or baseline survival at a given time point). | 18-19 |
|  | 15b | Explain how to the use the prediction model. | 18-19 |
| Model performance | 16 | Report performance measures (with CIs) for the prediction model. | 13-16 |
| Model-updating | 17 | If done, report the results from any model updating (i.e., model specification, model performance). | N/A |
| **Discussion** |  |  |  |
| Limitations | 18 | Discuss any limitations of the study (such as nonrepresentative sample, few events per predictor, missing data). | 21-22 |
| Interpretation | 19a | For validation, discuss the results with reference to performance in the development data, and any other validation data. | 19-21 |
|  | 19b | Give an overall interpretation of the results, considering objectives, limitations, results from similar studies, and other relevant evidence. | 19-21 |
| Implications | 20 | Discuss the potential clinical use of the prediction model and implications for future research. | 21-22 |
| **Other Information** |  |  |  |
| Supplementary information | 21 | Provide information about the availability of supplementary resources, such as study protocol, Web calculator, and data sets. | 18-19 |
| Funding | 22 | Give the source of funding and the role of the funders for the present study. | 25 |

**Notes:**

- N/A = Not applicable for this study design
- Page numbers refer to the manuscript pages where each item is addressed
- Some items may be addressed across multiple sections of the manuscript
